# Periodic Temperature Fluctuations as an Energy Source for RNA Evolution

**DOI:** 10.3390/life16060958

**Published:** 2026-06-05

**Authors:** Christian Mayer

**Affiliations:** Institute of Physical Chemistry, CENIDE, University of Duisburg-Essen, 45141 Essen, Germany; christian.mayer@uni-due.de; Tel.: +49-201-183-2570

**Keywords:** RNA world, temperature fluctuations, periodicity, thermodynamics, kinetics, driving force, energy source, molecular evolution, Carnot, heat engine

## Abstract

The effect of periodic temperature variation on short and partially matching interacting RNA strands is demonstrated using three pairs of competing RNA duplexes as simplified model systems. They represent random base-pairing interactions between short RNA chains at very early stages of RNA evolution. The molecular interaction kinetics are simulated based on the experimental thermodynamic data obtained by M. E. Christiansen et al. and on the Eyring theory. The simulated time developments demonstrate the impact of shifting reaction kinetics and a state of continuous non-equilibrium. The product mix that develops slowly at low temperatures releases chemical (free) energy quickly at high temperatures, and the product mix that develops quickly at high temperatures releases chemical (free) energy slowly at low temperatures. Regarding heat flow and energy storage, the chosen model system represents a generalized Carnot engine, similar to most comparable reactions during RNA evolution. Its action provides a perpetual driving force for selection processes, creating ideal conditions for an ongoing molecular evolution.

## 1. Introduction

The basic idea of RNA being the key polymer in the origin of life was initially proposed in the 1960s [1,2,3]. It was driven by the discovery that RNA strands, similar to proteins, can exhibit significant catalytic activity [4,5], including the ability to facilitate their own reproduction. These so-called ribozymes open the door to an efficient RNA-based self-sustaining molecular evolution, known as the RNA world [6]. Since its inception in 1986, this model has dominated a broader discussion on prebiotic evolution [6,7,8,9,10,11,12,13,14].

As postulated previously, the molecular evolution in the RNA world can be seen as a constant increase of order and complexity [15,16]. In this context, order and complexity are represented by the definition of RNA sequences and by their lengths, respectively. Each step of random formation increases complexity while partially reducing order. Each step of selection increases order while partially reducing complexity. Under suitable circumstances, a long sequence of such steps leads to a zig-zag course along a diagonal in an order/complexity diagram [16].

However, one essential question remains: what exactly is the driving force behind such a process, especially in its early stages? At this point, RNA chains would naturally be very short, with a catalytic effect being weak or missing completely. So how could longer chains have developed in the beginning?

The different ribozymes catalyzing the transcription of RNA strands that have been identified so far are quite demanding in their complexity [17,18,19,20,21]. In addition, a considerable sequential order is required, otherwise, the ribozymes catalyzing chain decomposition strongly compete against those that catalyze chain formation. Therefore, it cannot be expected that the beginning of this process is driven by self-reproduction. Instead, we have to assume a constant process of structural interaction between random short RNA strands that led to specific functions. These functions in turn could have served as efficient criteria for selection. We have to assume that functional structures may not have been particularly stable functions, therefore the driving forces for this process must have been beyond a simple search for thermodynamic stability.

The motivation of this article is the idea that the main driving force for the development of early functional RNA structures may have been provided by simple temperature fluctuations. The following study on simple model systems is meant to show that periodic temperature variations are a constant source of free enthalpy. In other words, oscillating temperatures can be seen as an energy source for the formation of RNA sequences beyond the state of thermodynamic equilibrium. Temperature fluctuations, e.g., on a daily basis may separate the conditions for formation from the conditions of selection, hereby defining the timeframe for consecutive steps leading to order and complexity.

## 2. An Elementary Model System: RNA Duplexes Containing Tandem Mismatches

Functional RNA structures such as the ribozymes for self-reduplication typically contain double strands, most of them being loops and containing partial mismatches [17,18,19,20,21]. Therefore, a suitable model system for functional RNA strands should at least reproduce some of these structural features. At the same time, it should be simple enough to allow for detailed thermodynamic studies. Some time ago, M.E. Christiansen and B.M. Znosko published profound thermodynamic studies on RNA duplexes containing tandem mismatches [22,23]. All these duplexes consist of ten base pairs with a tandem mismatch in the center [23]. The data are obtained by optical melting experiments on a temperature ramp with a heating rate of 1 K/min and with the absorbance measured at 280 nm [24,25]. Among a set of 33 duplexes listed in [23], three pairs of duplexes were chosen that fulfill the condition AB/AC; that is, one particular strand A is first combined with a partially matching strand B, forming the duplex AB, and then with another partially matching strand C to form the duplex AC. Then the thermodynamic data for the formation of AB are compared to those for the formation of AC, giving rise for the understanding of the particular competition of B and C for strand A. The thermodynamic data for the three pairs of duplexes extracted from [23] are listed in Table 1 (data obtained by an analysis of the melting curves are used due to their smaller experimental error).

**Figure 1:**
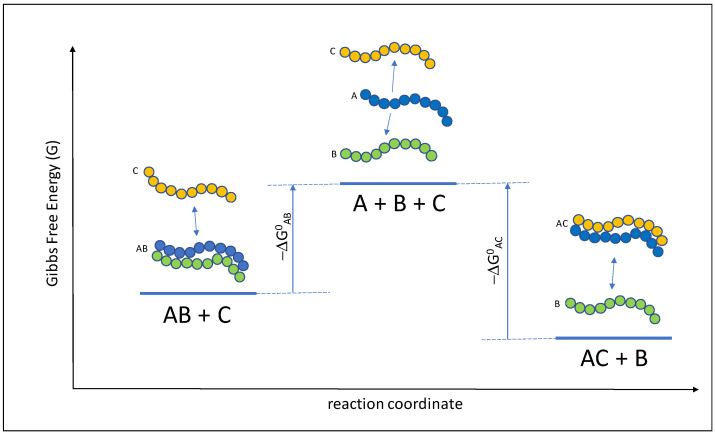
Mechanistic model for a single step of selection between two alternative RNA structures AB and AC. The alternative structures AB (left) and AC (right) consist of duplexes of ten base pairs with mismatches in the central part (see Table 1). The transition state in the center is represented by a set of separated strands A, B and C. In this representation, AC is depicted as the more stable structure but not necessarily the more functional one.

These data allow for a thermodynamic interpretation of molecular exchange between competing strands (Figure 1). Principally, the strands B and C compete for a common strand A in forming duplexes AB and AC. This competition and the resulting exchange reactions stand for a single step of selection in a molecular evolution process, where either the duplex AB or the duplex AC may be more functional. Hence, it is worthwhile to study the thermodynamics and kinetics of such a step in order to understand principal driving forces of the RNA world.

Of course, this mechanism with a single transition state is a simplifying approximation for a more complex procedure. It definitely does not hold for longer chains where unidirectional and bidirectional unzipping can take place [26]. However, in short hairpin structures formed by less than ten base pairs, the unzipping mechanism was found to be quite straightforward [27]. Further, the approach of this study neglects the mechanism of toehold-mediated RNA strand displacement [28,29]. This mechanism is certainly relevant for chains with longer matching sections where replacement can start on a toehold section and proceed in a step-by-step manner. However, for situations of random matching with a small number of base pairs and multiple mismatches at the beginning of the RNA world, the situation looks different. In this case, a duplex disintegration in a single step is much more likely. This finding is supported by data from the study of M.E Christiansen and B.M. Znosko on short RNA duplexes [23]. The very good match between thermodynamic parameters obtained by fitting individual melting curves with those derived from van’t Hoff plots (see Table 2 in [23]) is in favor of a simple one-step disintegration mechanism. In the following, the mechanistic interpretation depicted in Figure 1 will be used to derive an elementary understanding of the thermodynamics and kinetics of singular selection steps in the RNA world.

## 3. The Eyring–Polanyi Approach

According to the Eyring–Polanyi theory [30,31], a chemical reaction can be described in a multi-dimensional energy surface. The same approach has also been used to describe the viscosity of a nucleic acid solution with a given interaction between nucleic acid chains [32]. The reaction (or a single step in a flow process) follows the path of lowest energy, which then can be assigned to the reaction coordinate. The highest point along this pathway represents the transition state; the energy difference between the starting point and this transition state defines the activation energy. Introducing the free enthalpy as a measure for the energy and the quantum mechanical description of a vibration along the reaction coordinate and across the transition state (the Eyring vibration), Eyring and Polanyi derived an expression for the reaction rate constant k:

(1) k = κ k_B_ T h^−1^ exp(∆S^‡^/R) exp(−∆H^‡^/(RT))

where κ is the transmission coefficient, k_B_ is the Boltzmann constant, T is the absolute temperature, h is Planck’s constant, ∆S^‡^ is the activation entropy, R is the ideal gas constant and ∆H^‡^ is the activation enthalpy.

The Eyring theory can be applied to the reaction scheme in Figure 1 under the following conditions:

(1) The intermediate state A + B + C forms a saddle point on the Gibbs free energy surface of the exchange process. This is given since the free reaction enthalpy is negative in the forward as in the backward direction for all three cases within the given temperature range. In the first approximation, the state A + B + C may therefore be regarded as an activated complex, since every single step towards the educts or towards the products leads to a decrease in free energy. Obviously, it does not represent a real transitional state in terms of lifetime. Also, there will be multiple degrees of freedom for the course of the reaction from A + B + C towards the reaction products. Presumably, the pre-exponential factor in Equation (1) describes the upper limit of the reaction rates as described by a vibrational mode. However, a common correction factor κ will easily account for a corresponding rate reduction in both directions.
(2) The state A + B + C is in a special kind of equilibrium, the so-called quasi-equilibrium, with AB + C as well as with AC + B. The special feature of this state is that two separate populations of A + B + C are considered: one population that is part of the forward reaction, and one population that is part of the backward reaction. In this model, the flux of the forward reaction is independent of the flux in the backward reaction. Overall, the total equilibrium situation still follows the common rules of thermodynamics. Most importantly, the equilibrium between AB + C and A + B + C and the one between A + B + C and AC + B are being accounted for at all times.
(3) The activated complex A + B + C can convert into AB + C as well as into AC + B, and both conversions follow the rules of kinetic theory. The overall rate for the conversion of AB + C into AC + B (and vice versa) now includes the equilibrium constant for the formation of the transition state as well as a kinetic constant for the conversion of the transition state into the product. Therefore, it combines reaction kinetics with reaction thermodynamics.

Under these conditions and following the reaction scheme in Figure 1, the rate constant k_1_ for the forward reaction from AB + C towards AC + B writes as follows:

(2) k_1_ = κ k_B_ T h^−1^ exp(−∆S^0^_AB_/R) exp(∆H^0^_AB_/(RT))

where ∆S^0^_AB_ and ∆H^0^_AB_ are the standard entropy and the standard enthalpy for the formation of AB according to Table 1. The signs of both values are now inverted as the initial steps towards the activated complex consist of duplex separation instead of duplex formation. Correspondingly, a similar expression is derived for the reaction from AC + B towards AB + C:

(3) k_2_ = κ k_B_ T h^−1^ exp(−∆S^0^_AC_/R) exp(∆H^0^_AC_/(RT))

In these equations for k_1_ and k_2_, all parameters are now clearly defined, with the exception of the transmission coefficient κ. It accounts for the probability of the reactants to actually form the products once the transition state is overcome. In the ideal case (like in the original Eyring equation), it is set to unity [30,31]. However, in some other cases, the reactants may become partially trapped in an intermediate position, leading to a transmission coefficient smaller than 0.5 [33]. Such a phenomenon may possibly occur in the reaction from AB + C to AC + B and very likely in the opposite direction as well. Hence, one can expect a certain scaling factor κ for the rate constants k_1_ and k_2_. However, this does not affect the resulting equilibrium state, since very similar reaction pathways are expected for both directions. Instead, it simply scales the reaction rates to a minor extent (as we deal with changes over several orders of magnitude). In the following, all transmission coefficients κ are set to unity in the first approximation, just as in the original treatment by Eyring and Polanyi.

## 4. Time Development of Duplex Concentration

Based on Equations (2) and (3), the course of the reaction between AB + C and AC + B may be numerically simulated. Following common rules of reaction kinetics, the rates for the formation of the duplexes AB and AC (and for the single chains C and B) are given by

(4) dc_AB_/dt = dc_C_/dt = −k_1_ c_AB_ c_C_ + k_2_ c_AC_ c_B_

and

(5) dc_AC_/dt = dc_B_/dt = −k_2_ c_AC_ c_B_ + k_1_ c_AB_ c_C_

Introducing discrete time steps ∆t, the stepwise development of Equations (4) and (5) lead to the following approximations:

(6) c_AB_(t + ∆t) ≅ c_AB_(t) − k_1_ c_AB_(t) c_C_(t) ∆t + k_2_ c_AC_(t) c_B_(t) ∆t

(7) c_C_(t + ∆t) ≅ c_C_(t) − k_1_ c_AB_(t) c_C_(t) ∆t + k_2_ c_AC_(t) c_B_(t) ∆t

(8) c_AC_ (t + ∆t) ≅ c_AC_(t) − k_2_ c_AC_(t) c_B_(t) ∆t + k_1_ c_AB_(t) c_C_(t) ∆t

(9) c_B_ (t + ∆t) ≅ c_B_(t) − k_2_ c_AC_(t) c_B_(t) ∆t + k_1_ c_AB_(t) c_C_(t) ∆t

With a given variation of the temperature T(t), the rate constants k_1_ and k_2_ become time dependent as well, according to Equations (2) and (3). Consequently, variables k_1_ and k_2_ in Equations (6)–(9) have to be replaced by k_1_(t) and k_2_(t). The simulation begins at t = 0 with a set of given starting concentrations c_AB_(0), c_C_(0), c_AC_(0), and c_B_(0) at a starting temperature T(0). Initially, the time interval ∆t is systematically shortened until the variation between the subsequent test runs is negligible. Under these conditions, a reliable time development of c_AB_(t), c_C_(t), c_AC_(t), and c_B_(t) at a given temperature/time function T(t) is obtained.

Based on the time-dependent concentrations, the time-dependent enthalpy state of the mixture is calculated to determine the heat flow into and out of the system. For convenience, the enthalpy of the transition state A + B + C is set to zero. The relative enthalpy H_rel_ of a given solution volume then results as

(10) H_rel_(t) = ∆H^0^(AB) ∙ c_AB_(t) + ∆H^0^(AC) ∙ c_AC_(t)

In the first approximation, all standard enthalpy values are assumed to be temperature independent within the given temperature window.

## 5. Results: Model Calculations

Assuming a much faster planetary rotation, a much shorter day/night periodicity under prebiotic conditions on the early Earth is reasonable. For the following calculations, we choose a daily period of 10 h which may have occurred some 4 billion years ago [34]. In addition, the early Earth is thought to have had moderate temperatures, similar to those experienced today [35]. Among the possible variety of temperature conditions around a primordial pond, we choose an ideal setting. During the course of a single day, we assume a corresponding sinusoidal temperature variation between T_min_ = 275 K (right above the melting point of water) and a temperature T_max_, which is set 1 K below the lowest melting point of the two duplexes AB and AC. Full melting of RNA duplexes is avoided under ideal conditions, since single chains are more susceptible to hydrolysis. With ∆T = (T_max_ − T_min_)/2 and T_av_ = T_min_ + ∆T, and with ω = 2 π 0.1 h^−1^, the time-dependent temperature becomes

(11) T(t) = T_av_ + ∆T sin(ωt)

For simplicity, all calculations start with identical concentrations for all specimens, such that c_start_ = c_AB_(0) = c_C_(0) = c_AC_(0) = c_B_(0). During the exchange process indicated in Figure 1 and following Equations (1)–(9), the concentrations for AB and C start to differ from those for AC and B (while c_AB_(t) = c_C_(t) and c_AC_(t) = c_B_(t) at all times due to the stoichiometry of the reaction).

**Figure 2:**
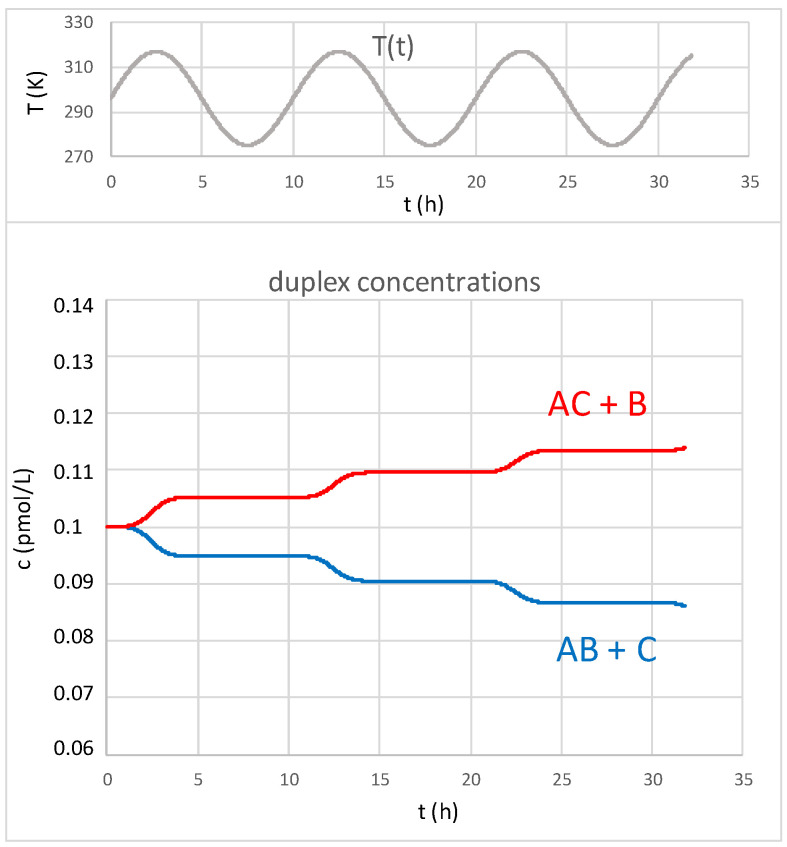
Time development of the concentrations of AB(1)/C(1) (blue) and AC(1)/B(1) (red) under the influence of a periodic temperature oscillation (top) between 275 and 316.85 K (1 K below the melting point of AB(1)) for very low starting concentrations (c_start_ = 0.1 pmol/L).

Initially, the study focuses on the variation of the starting concentrations c_start_. Thermodynamic data are chosen according to case 1 in Table 1, such that the exchange between AB(1) and AC(1) is being observed. Figure 2 shows the time development of AB(1)/C(1) and AC(1)/B(1) under the influence of a periodic temperature oscillation between 275 and 318 K for very low starting concentrations (c_start_ = 0.1 pmol/L).

Due to the larger (negative) free enthalpy of AC(1), the development is in favor of the products AC and B. Clearly, the concentrations do not reach the equilibrium state during the given time window of 32 h. With a starting concentration corresponding to approximately 600,000 molecules per cubic millimeter (or an average distance between adjacent molecules of 11.85 μm), the RNA chains need considerable time just to find each other. The reaction rates, as reflected by the slopes of the concentration plots, are strongly influenced by temperature and follow the Arrhenius law. Within the given temperature range, the reaction rates vary by more than six orders of magnitude, resulting in alternating steep and shallow sections of the plot.

**Figure 3:**
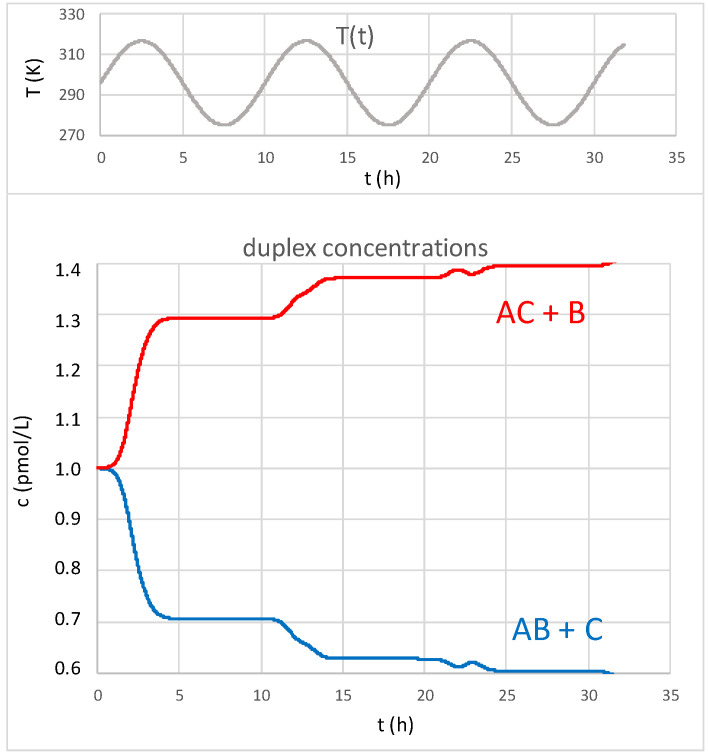
Time development of the concentrations of AB(1)/C(1) (blue) and AC(1)/B(1) (red) under the influence of a periodic temperature oscillation (top) between 275 and 316.85 K (1 K below the melting point of AB(1)) for a starting concentration of c_start_ = 1 pmol/L.

An increase of the starting concentration by one order of magnitude (c_start_ = 1 pmol/L) already leads to a significantly different development (Figure 3). Both plots now show some asymptotic behavior, visibly approaching the equilibrium concentrations. In addition, some small “wiggles” appear at t = 22 h during the peak temperatures near T_max_.

**Figure 4:**
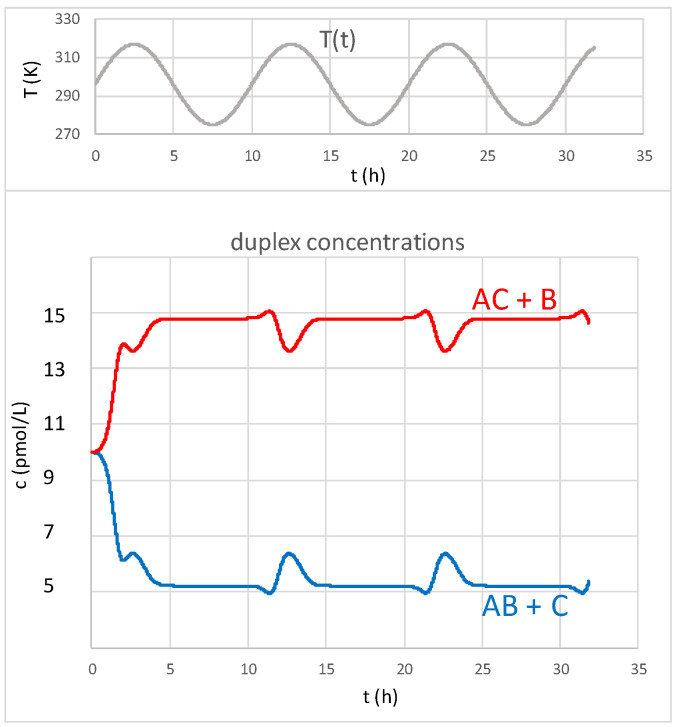
Time development of the concentrations of AB(1)/C(1) (blue) and AC(1)/B(1) (red) under the influence of a periodic temperature oscillation (top) between 275 and 316.85 K (1 K below the melting point of AB(1)) for a starting concentration of c_start_ = 10 pmol/L.

A starting concentration of 10 pmol/L leads to a rapid development towards the equilibrium and to a much stronger expression of the “wiggles” (Figure 4). These now clearly indicate that higher temperatures not only increase the reaction rates but also influence the equilibrium constant, somewhat reducing the concentration of AC and B in accordance with Le Chatelier’s principle: The formation of AC(1) is more exothermic than the one of AB(1), see Table 1.

**Figure 5:**
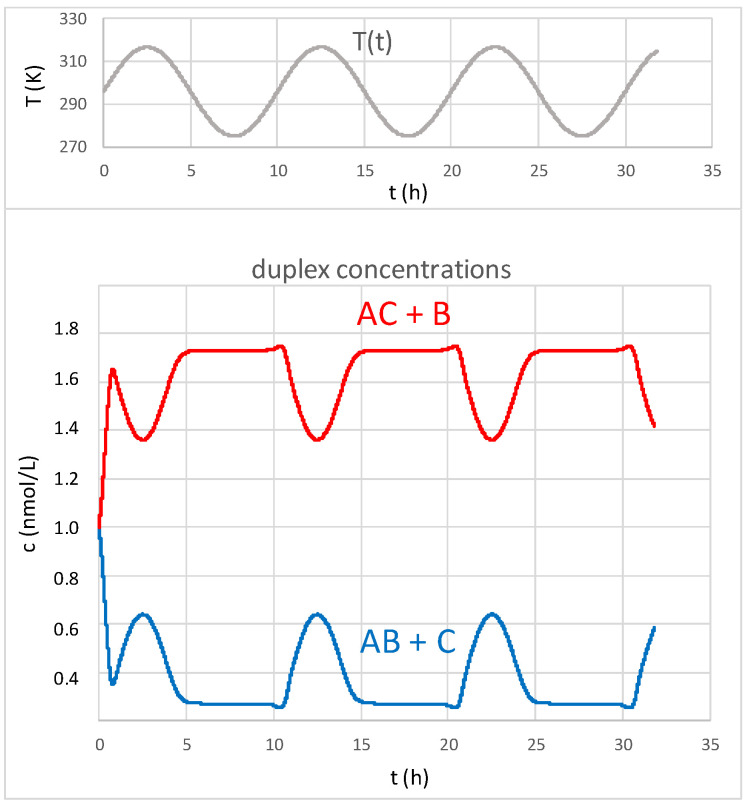
Time development of the concentrations of AB(1)/C(1) (blue) and AC(1)/B(1) (red) under the influence of a periodic temperature oscillation (top) between 275 and 316.85 K (1 K below the melting point of AB(1)) for a starting concentration of c_start_ = 1 nmol/L.

Finally, at a starting concentration of 1000 pmol/L (c_start_ = 1 nmol/L), the temperature-induced oscillations of the equilibrium constant dominate the development (Figure 5).

The equilibrium state for low temperatures would put the products AC + B even more strongly in favor. The formation of AC(1) is more exothermic than the formation of AB(1); therefore, it would have an advantage according to Le Chatelier’s principle. However, this low-temperature equilibrium is not reached within a five-hour period due to the given slow reaction rates. At high temperatures, in turn, the advantage of AC + B is significantly reduced, which quickly is apparent with the given rapid kinetics. Together, these effects are responsible for the peculiar shape of the concentration curves (“wiggles”).

**Figure 6:**
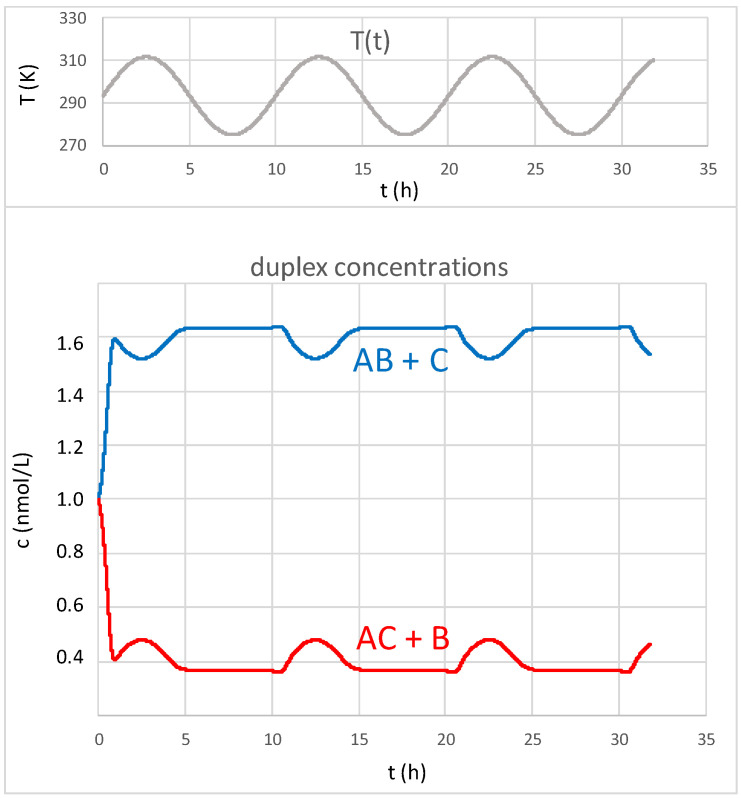
Time development of the concentrations of AB(2)/C(2) (blue) and AC(2)/B(2) (red) under the influence of a periodic temperature oscillation (top) between 275 and 311.75 K (1 K below the melting point of AC(2)) for a starting concentration of c_start_ = 1 nmol/L.

Finally, a comparison should be made for the three duplex varieties, (1), (2) and (3), according to Table 1. The results for the duplex competition AB(2)/C(2) and AC(2)/B(2) is shown in Figure 6. Looking at the corresponding enthalpy values, it is obvious that the reaction enthalpy for the exchange is quite small (∆H = ±10 kcal/mol, depending on the direction). This necessarily leads to much smaller oscillations of the equilibrium state. In this case, the duplex AB is favored against AC. Due to the lower melting temperature of AC(2), the peak temperature T_max_ has to be reduced to 311.75 K.

**Figure 7:**
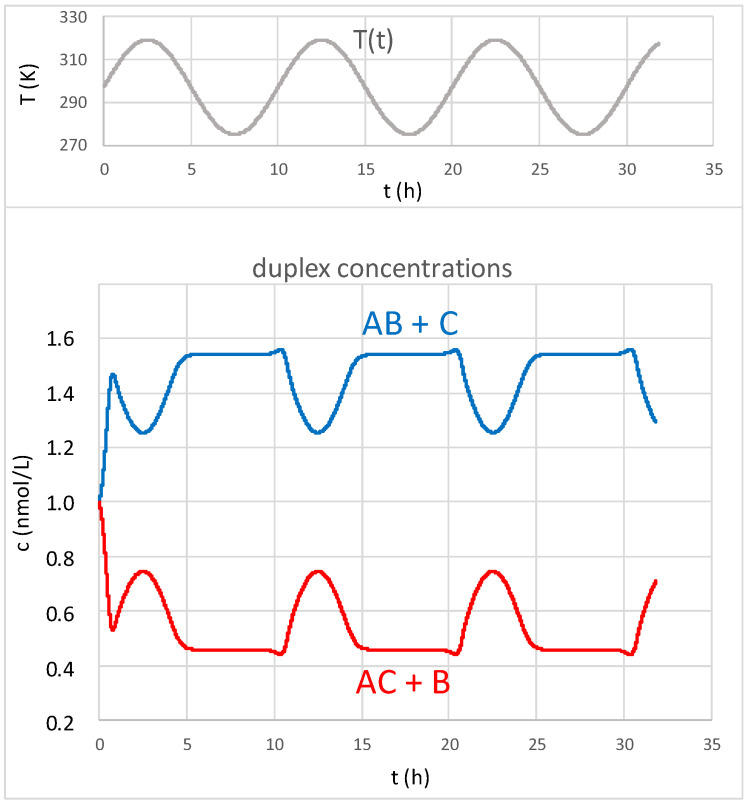
Time development of the concentrations of AB(3)/C(3) (blue) and AC(3)/B(3) (red) under the influence of a periodic temperature oscillation (top) between 275 and 319.35 K (1 K below the melting point of AC(3)) for a starting concentration of c_start_ = 1 nmol/L.

The results for the duplex competition AB(3)/C(3) and AC(3)/B(3) again resembles case (1), except that the duplex AB is thermodynamically favored (Figure 7). The formation enthalpy from AC + B amounts to ∆H = −16.5 kcal/mol leads to an oscillation almost as strong as in case (1). Due to higher melting temperatures, T_max_ can be re-adjusted to 319.35 K. Again, the low-temperature equilibrium is not reached due to slow kinetics, while the high-temperature equilibrium is rapidly achieved.

## 6. Discussion

### 6.1. General Approach of the Model

Clearly, base-pairing interactions are crucial for function and selection in the RNA world. They play an essential role in temporary interactions between RNA chains, in the formation of temporary or permanent internal loops, and in reduplication and catalytic functions. Therefore, an understanding of their thermodynamics and kinetics is fundamental to evaluate processes that may have led to RNA evolution.

Using competing duplexes with partial mismatches as a model system for (early) RNA base-pairing interactions has specific advantages: it provides a realistic example of chain–chain interactions and, in terms of its basic mechanism, is quite similar to the reversible formation of an internal loop. It incorporates both local base pairing and the entropic effects of chain motion. Overall, its thermodynamics and kinetics mirror crucial steps in the postulated early steps of RNA evolution.

On the surface of early Earth, oscillating temperature conditions likely occurred on a daily basis with a period of approximately 10 h [34]. The average temperatures and temperature amplitudes may have been similar to those observed today, depending on local conditions [35]. Overall, average temperatures of around 300 K and daily temperature variations of more than 20 K seem reasonable, especially under the influence of direct sunlight. Obviously, the temperature plays an important role in molecular evolution. Particularly, the influence of temperature on a system of interacting RNA strands has two aspects: it affects the reaction rate (kinetics) and the position of equilibrium (thermodynamics). When the temperature is constant, the van’t Hoff equation determines the (constant) equilibrium state while the Arrhenius law determines the (constant) equilibration rate. However, under oscillating temperature conditions, the situation becomes much more complicated: the equilibrium position shifts permanently, and the reaction rates vary significantly over time.

In this study, the effect of oscillating temperatures has been simulated for competing duplexes formed by three RNA chains, A, B, and C, as model systems. Based on the thermodynamic data obtained by M.E. Christiansen and B.M. Znosko [23], the exchange reaction between the states AB + C and AC + B could be analyzed using the Eyring–Polanyi theory. Three different sets of RNA chains, A, B, and C, were included, accounting for slightly different base sequences. The results show the time developments of the concentrations of AB/C and AC/B for all three cases under the influence of oscillating temperatures.

### 6.2. The Influence of the Chain Concentration

At the beginning of a random RNA chain formation process, starting concentrations may have been very low. Under these conditions, the pairing competition of independent chains remains quite far from the equilibrium state, with reaction rates periodically accelerating during peak temperatures (see Figure 2 and Figure 3). Step by step and over longer periods of time, the thermodynamically favored pairings (AC(1), AB(2), and AB(3)) become dominant over their alternatives. However, the kinetics of their formation is just too slow to develop a fruitful resonance with the daily temperature oscillation. The development is limited to a simple asymptotic approach towards equilibrium with fluctuating velocity. There is neither a significant energy transfer nor an effect on the driving force. In contrast, the situation changes drastically at concentrations above 10 pmol/L. This is the actual starting point of the energy transfer.

### 6.3. Temperature Fluctuations as a Source of Energy

**Figure 8:**
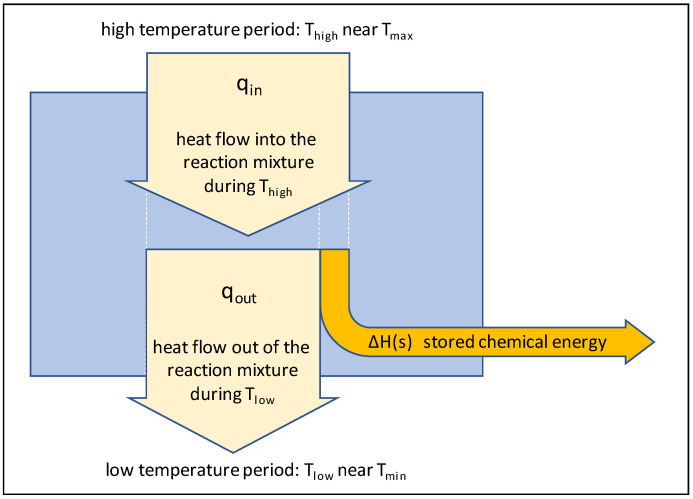
Schematic representation of the energy transfer during temperature cycling.

Does an oscillating temperature actually deliver energy into the system? How can a simple variation of an intensive state parameter induce energy flow? The answer is that the system acts as a heat engine, following the generalized Carnot principle (Figure 8). It takes up a larger amount of heat q_in_ at high temperatures (T_high_ near T_max_) and releases a smaller amount of heat q_out_ at low temperatures (T_low_ near T_min_). The difference between the two quantities q_in_ and q_out_ is used to produce high-energy products representing stored chemical energy ∆H(s). Their high-energy state is then kinetically locked: high-energy compounds are partially preserved due to the slower kinetics at lower temperatures.

In order to fulfill the second law of thermodynamics, |q_in_|/T_high_ must be slightly smaller than |q_out_|/T_low_, such that the overall entropy change of the system is positive. However, since T_high_ > T_low_, this leaves a possible difference |q_in_| − |q_out_| that can be transformed into mechanical or chemical energy. The overall efficiency η of this energy gain, i.e., the relation between |ΔH(s)| and |q_in_|, is limited by the Carnot formula η ≤ (T_high_ − T_low_)/T_high_. In the given model, this relation is thermodynamically introduced by the van’t Hoff equation. For our RNA model process at T_high_ − T_low_ = 31.85 K, the theoretical Carnot efficiency amounts to 10.2%.

**Figure 9:**
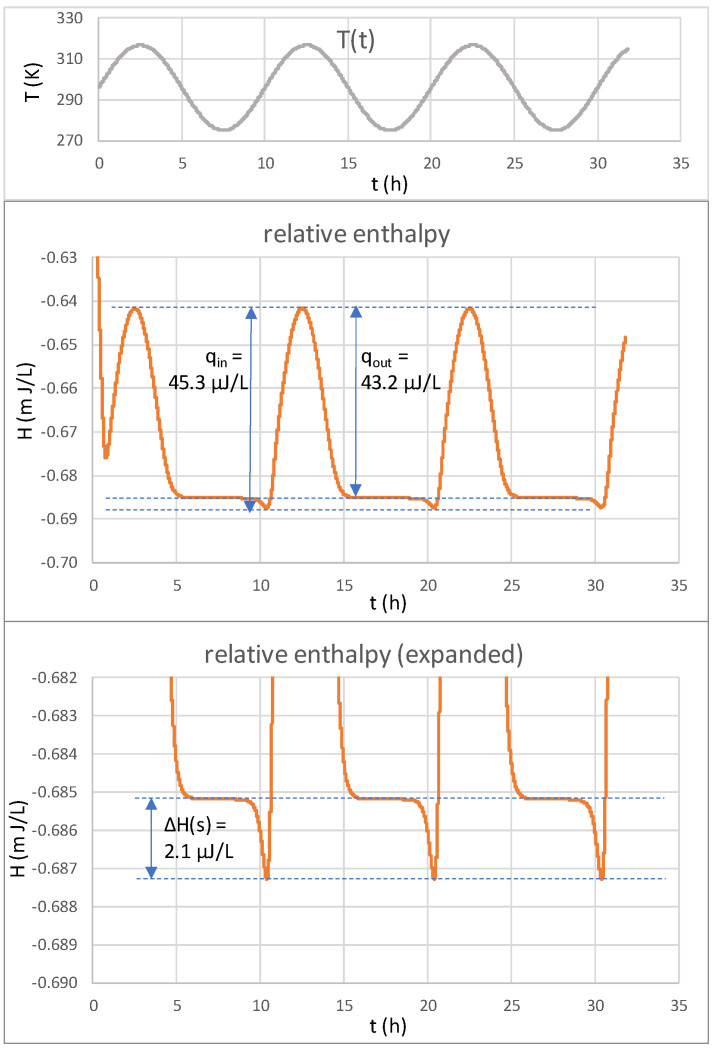
Development of the system enthalpy over time, calculated for AB(1)/C(1) and AC(1)/B(1) under the influence of a periodic temperature oscillation between 275 and 316.85 K for a starting concentration of c_start_ = 1 nmol/L (see Figure 5 for comparison). Enthalpy values are given in relation to H(A + B + C), which is arbitrarily set to zero. During the high-temperature phase, a heat uptake of 45.3 µJ/L is observed (**center**). During the low-temperature phase, 43.2 µJ/L is emitted. The residual quantity of 2.1 µJ/L is kinetically locked in the system as stored chemical energy and will only be released at the beginning of the next heat cycle (**bottom**).

Figure 9 shows the development of the system enthalpy over time, calculated for the data plotted in Figure 5 according to Equation (10). The time-dependent enthalpy values refer to the development of the concentrations of AB(1)/C(1) and AC(1)/B(1) under the influence of a periodic temperature oscillation (top) between 275 and 316.85 K for a starting concentration of c_start_ = 1 nmol/L. At the beginning of the high-temperature phase (T_high_ ≥ T_max_ − 5K), the reaction system takes up a total of 45.3 µJ/L of heat (Figure 9, center). At the end of the high-temperature phase, it releases a total of 43.2 µJ/L of heat. The difference of 2.1 µJ/L is transformed into stored chemical energy, kinetically locked in the low-temperature phase (T_low_ ≤ T_min_ + 5K). It is only released shortly before the next high-temperature cycle starts (Figure 9, bottom). In a more complex chemical system, it could also be used as chemical energy for a secondary reaction. This is actually the amount of energy being harvested in a single cycle of the given Carnot-like heat engine.

The actual efficiency of this process Is given as η = (2.1/45.3), which corresponds to 4.6%. Compared to the theoretical Carnot efficiency of 10.2%, this means that more than half of the available energy is being lost. This mainly results from the imperfect kinetic “freezing” during the low-temperature phase. Nevertheless, 4.6% of the total heat uptake is a considerable amount of energy that is directly available to other connected chemical steps. In case of catalysis (which is quite typical for RNA strands), this could power an uphill process, long before the non-productive energy release is triggered by increasing temperature.

### 6.4. Temperature Fluctuations as a Driving Force for RNA Evolution

Stored chemical energies as detected by enthalpy changes are only part of the driving force of a reaction system. Driving forces depend on free enthalpy changes ΔG and are directly observable in the course of the reaction. In the simulated reaction plots, this becomes evident at concentrations above 10 pmol/L where the time development is predominantly influenced by the variation of the equilibrium state (Figure 4, Figure 5, Figure 6 and Figure 7). While the low-temperature equilibrium is not reached due to the given slow reaction rates, the high-temperature equilibrium is quickly achieved. The product mix that develops slowly at low temperatures releases chemical free energy quickly at high temperatures, while the product mix that develops quickly at high temperatures releases chemical free energy slowly at low temperatures. Together, these effects lead to distinctive peaks in the time development of the various duplex concentrations (Figure 4, Figure 5, Figure 6 and Figure 7), resulting in plots that resemble a sequence of pulses. The intensity of these pulses largely depends on the absolute reaction enthalpy difference between AB and AC. A comparison of cases (1) to (3) shows that pulses are strongest for case (1), with ∆H^0^(AB) − ∆H^0^(AC) = 118.0 kJ, and smallest for case (2), with ∆H^0^(AB) − ∆H^0^(AC) = −41.8 kJ.

This pulse-like behavior marks a very important outcome of the simulation. Due to the ideal temperature oscillations, the competing-chain system is periodically disturbed and forced into new pairing situations. Dead-end products are efficiently avoided. Complex structures of associated RNA never fully melt, thereby slowing down the hydrolysis of RNA chains. Oscillating temperatures keep the system outside of equilibrium permanently, resulting in a substantial driving force for multiple restructuration pathways. Even though this driving force is thermodynamic in origin, this does not mean that it produces the most thermodynamically stable products. In fact, the interaction between thermal pushing and the low-temperature kinetic trap that occurs during temperature cycling practically avoids thermodynamic stability. This cooperation between kinetics and thermodynamics provides the basis for the concept of *dynamic kinetic stability* (DKS) [36]. Even if the process is not yet replicating, it is still governed by both kinetics and thermodynamics, and it never falls into the trap of thermodynamic stability. If the process eventually leads to replication, it can further progress to a situation where the persistence principle [37] becomes relevant. In this case, selection is largely dominated by replication efficiency.

Without temperature oscillations, the product concentrations would simply level out over the course of a reaction, driven by a temporary reservoir of free enthalpy G and asymptotically approaching dG = 0. With temperature oscillations, however, this reservoir would repeatedly fill up, effectively providing an eternal source of free enthalpy, with the chemically stored enthalpy forming a strong contribution. Regarding the enthalpy, temperature oscillations may have been the simplest and probably the earliest energy source for prebiotic reactions. From a thermodynamic perspective, it is a source of energy comparable to a Carnot engine. Although its efficiency may be relatively low, the resulting enthalpy gain is delivered repetitively and does not fade.

Furthermore, alternating periods of low and high temperatures could separate subsequent steps of formation and selection, thereby driving the evolutionary process towards order and complexity [16]. Overall, oscillating temperature conditions are expected to play a pivotal role in the successful evolution of RNA.

## Figures and Tables

**Table 1:** Thermodynamic data for the formation of three pairs of duplexes AB/AC as reported by M.E. Christiansen and B.M. Znosko [23]. Note that some sequences are inverted with respect to [23] in order to correspond to the duplex identities AB and AC. As in the original source, standard reaction enthalpies ∆H^0^ are given in kcal/mol, standard reaction entropies ∆S^0^ in entropy units (cal/(K∙mol)) and melting temperatures T_M_ in °C. All data were derived by fitting melting curves to a two-state model [23].

Strand Identity	Sequence	−∆H^0^ (kcal/mol)	−∆S^0^ (eu)	T_M_ (°C)
AB(1)	_5′_GACU^AA^GCUG_3′ 3′_CUGA_GG_CGAC_5′_	57.5 ± 8.1	159.7 ± 25.8	44.7
AC(1)	_5′_GACU^AA^GCUG_3′ 3′_CUGA_AG_CGAC_5′_	85.7 ± 3.1	245.7 ± 9.9	48.0
Strand identity	Sequence	−∆H^0^ (kcal/mol)	−∆S^0^ (eu)	T_M_ (°C)
AB(2)	_3′_CUGU^AG^UGAC_5′ 5′_GACA_GA_GCUG_3′_	82.4 ± 7.4	238.0 ± 23.5	45.0
AC(2)	_3′_CUGU^AG^UGAC_5′ 5′_GACA_AA_GCUG_3′_	72.4 ± 3.7	210.5 ± 12.1	39.6
Strand identity	Sequence	−∆H^0^ (kcal/mol)	−∆S^0^ (eu)	T_M_ (°C)
AB(3)	_3′_CUGC^AG^UGAC_5′ 5′_GACG_GA_GCUG_3′_	77.5 ± 5.2	218.9 ± 16.0	50.0
AC(3)	_3′_CUGC^AG^UGAC_5′ 5′_GACG_AA_GCUG_3′_	61.0 ± 4.1	169.3 ± 12.9	47.2

## Data Availability

All numeric data are archived by the author.
